# Effectiveness of labels in digital art experience: psychophysiological and behavioral evidence

**DOI:** 10.3389/fpsyg.2024.1342667

**Published:** 2024-07-01

**Authors:** Serena Castellotti, Ottavia D’Agostino, Maria Michela Del Viva

**Affiliations:** Department of Neurofarba, University of Florence, Florence, Italy

**Keywords:** neuroaesthetic, artworks labels, digital art, psychophysiology, museum vs. laboratory setting, pupillometry

## Abstract

**Introduction:**

Nowadays museums make large use of digital materials (e.g., virtual tours) to attract visitors. Therefore, it is worthwhile investigating which variables affect the engagement with art outside the museum, and whether digital reproductions of artworks are as effective as museum originals in producing a satisfying aesthetic experience.

**Methods:**

Here we tested the effectiveness of introducing additional informative materials on the artistic enjoyment of contemporary paintings presented on a computer screen. Naïve observers were exposed to essential and descriptive labels before viewing artworks. We flanked traditional measurement methods - viewing times and questionnaires, with biometric parameters – pupil responses, eye movements, heart rate, and electrodermal activity. The results were then compared to our previous museum study that adopted the same experimental paradigm.

**Results:**

Our behavioral and psychophysiological data lead to a complex pattern of results. As found in the museum setting, providing detailed descriptions decreases complexity, evokes more positive sensations, and induces pupil dilation but does not enhance aesthetic appreciation. These results suggested that informative labels improve understanding and emotions but have a limited impact on the hedonic evaluation of artworks in both contexts. However, other results do not mirror those found in the museum; in the laboratory setting, participants spend a similar amount of time, have a comparable gaze behavior, and their electrodermal activity and heart rate do not change when viewing artworks with different types of labels. The main difference between the lab and museum settings is the shorter time spent viewing digital reproductions vs. real paintings, although subjective ratings (e.g., liking, interest) are comparable.

**Discussion:**

Overall, this study indicates that the environmental context does impact the aesthetic experience; although, some beneficial effects of introducing additional relevant content in labels accompanying artworks can also be acquainted through digital media outside of the museum.

## 1 Introduction

Neuroaesthetics, originally dedicated to exploring neural bases underlying art perception (Zeki and Lamb, 1994), has evolved to investigate how art impacts human cognition, emotions, and behavior (Pearce et al., 2016; Magsamen, 2019). Recent years have witnessed extensive research into the factors influencing aesthetic experience (Pes et al., 2012; Packer and Ballantyne, 2016), encompassing the characteristics of the artwork itself (e.g., size, abstraction, balance, and symmetry), the viewer (e.g., age, expertise, and personality traits), and the physical context of the exhibition (e.g., lighting and art labels; for a review, see Pelowski et al., 2017).

Traditionally, the quality of the aesthetic experience has been assessed through self-report questionnaires or post-visit interviews (Cupchik et al., 1994; Nadal et al., 2010; Mastandrea et al., 2011; Wanzer et al., 2018). Some studies also exploit behavioral measurements, such as tracking of visit pathways (Smith and Smith, 2001; Brieber et al., 2014) or recording of dwell times (Locher et al., 2001; Carbon, 2017; Smith et al., 2017; Szubielska et al., 2021b). Viewing times that visitors dedicated to artworks, being associated with visual attention and cognitive processes, can serve as indicators of learning and interest. Utilizing advanced tools such as eye-tracking and devices for physiological data recordings, researchers can now obtain other objective and real-time parameters that characterize the human aesthetic experience (e.g., Tröndle et al., 2012; Tschacher et al., 2012; Rainoldi et al., 2018, 2020; Di Giovanni, 2020; Estrada-Gonzalez et al., 2020; Garbutt et al., 2020; Reitstätter et al., 2020; Castellotti et al., 2023). These biometric measurements include respiratory rate, temperature, heart rate (Tschacher et al., 2012; Ménard et al., 2015; Xiefeng et al., 2019; Bulagang et al., 2020), skin responses (Hot et al., 2005; Sequeira et al., 2009; Tschacher et al., 2012; Ménard et al., 2015), gaze behavior (Kardan et al., 2015; Estrada-Gonzalez et al., 2020), pupil response (Bradley et al., 2008; Binda and Murray, 2015; Joshi and Gold, 2020), and more, all of which reflect emotional and cognitive processes.

Laboratory research has made significant contributions to our understanding of the factors influencing the aesthetic experience (e.g., Nadal et al., 2010; Mastandrea et al., 2011; Gernot et al., 2018; Darda and Cross, 2022). Many of these found that non-expert people usually prefer representational paintings compared to abstract ones (Cupchik et al., 1994; Mastandrea et al., 2007, 2009; Pihko et al., 2011; van Paasschen et al., 2015; Bubić et al., 2017; Szubielska et al., 2018, 2021b; Bimler et al., 2019), possibly because abstract artworks often convey ambiguity, making them challenging to interpret compared to the clarity of representational art (Kettlewell et al., 1990; Bimler et al., 2019). In laboratory environments, it has also been found that the pupil diameter (Kuchinke et al., 2009; Castellotti et al., 2020, 2021) and eye movements (Massaro et al., 2012; Francuz et al., 2018; Fudali-Czyz et al., 2018; Estrada-Gonzalez et al., 2020) can serve as indicators of individual reactions to artworks. For example, some studies delve into the concept of *processing fluency*, which pertains to how easily information is processed by the human mind, and found that paintings with high processing fluency, leading to higher preference (*hedonic fluency model*; see Reber et al., 2004; Smith and Smith, 2006), were also associated with increased pupil dilation (Kuchinke et al., 2009), interpreted as reflecting aspects of aesthetic emotions (Leder et al., 2004).

Several studies found that art experts exhibit longer saccades and fewer, shorter fixations on salient areas when viewing paintings compared to non-expert observers (Pihko et al., 2011; Koide et al., 2015; Francuz et al., 2018; Sharvashidze and Schütz, 2020; Song et al., 2021). Finally, some studies investigated how aesthetic preference is modulated by familiarity (and novelty), however yielding inconsistent results (cf., Leder, 2001; Mikuni et al., 2022).

For the main point of the current work, we focus on the studies testing the effects of labels on the enjoyment of artworks (Serrell, 1996). A significant debate exists among art curators between those supporting the utility of titles and texts in enhancing the observers’ experience and those claiming that artworks should be “self-explanatory” (Pekarik, 2004; Trione, 2017, 2023; Sitzia, 2018; Conti, 2022). The *stage model of aesthetic processing* claims that information provided to viewers helps them understand and consequently affects their esthetic judgments (Leder et al., 2004). In fact, numerous laboratory studies demonstrated that titles and explanations of artworks contribute to a better understanding of their content (Cupchik et al., 1994; Russell and Milne, 1997; Millis, 2001; Leder et al., 2006; Bubić et al., 2017; Lin and Yao, 2018; Szubielska et al., 2018) and a greater appreciation (Russell, 2003; Swami, 2013; Gerger and Leder, 2015; Lin and Yao, 2018; Mullennix and Robinet, 2018; Bailey-Ross et al., 2019; Ganczarek et al., 2022).

Although laboratory studies have provided valuable insights, recent research underscores the significance of examining the aesthetic experience within its ecological context. Museum studies typically rely on the observation of visitors’ behavior in free-choice conditions (Brieber et al., 2014, 2015a; 2015b; Tröndle et al., 2014; Carbon, 2017) and post-visit questionnaires (Mastandrea et al., 2007, 2009; Brieber et al., 2014; Tröndle et al., 2014; Carbon, 2017; Skov et al., 2018). Recently the use of mobile eye-tracking (MET) and other wearable wireless devices in museums has also become increasingly popular because they enable the measurement of attention and various bio-signals in a non-intrusive manner in real-life settings (e.g., Tröndle et al., 2012; Tschacher et al., 2012; Brieber et al., 2015b; Walker et al., 2017; Pelowski et al., 2018; Rainoldi et al., 2018, 2020; Scott et al., 2019; Di Giovanni, 2020; Garbutt et al., 2020; Reitstätter et al., 2020; Kühnapfel et al., 2023). Again, among these museum-based research, those focusing on informative materials are of particular importance for our research interests (Rainoldi et al., 2018; Reitstätter et al., 2020, 2022; Szubielska et al., 2021b; Szubielska and Imbir, 2021). For example, it has been found that the introduction of new information in an exhibition, leads visitors to spend more time engaging with the artworks (Reitstätter et al., 2020, 2022). These exploratory studies offer the advantage of gathering large sample sizes by involving regular museum visitors. Moreover, they ensure a naturalistic experience as visitors navigate through the exhibition at their own preferences, occasionally accompanied by others (Smith and Smith, 2001; Carbon, 2017; Smith et al., 2017). However, these studies do not focus on providing detailed measurements of the psychophysiological processes occurring in each observer when viewing artworks in response to specific variables like museum-provided labels.

Recently, our research group investigated how descriptive labels influence the enjoyment of artworks in a contemporary art museum (Castellotti et al., 2023). Employing a structured experimental protocol, although diverging from the mentioned ecological conditions, allowed us to control confounding variables and obtain reproducible and accurate measurements of multiple physiological and behavioral parameters. We found that a detailed description (compared to an essential one) leads observers to spend more time looking at the artwork, to feel more positive emotions, and to judge the piece of art as less complex, while their bodies react by increasing electrodermal activity and dilating pupillary diameter (Castellotti et al., 2023).

Before delving into our current study, it is important to acknowledge the growing role of the digital world in art consumption. Indeed, nowadays museums use more and more digital material to attract visitors, such as online exhibitions, virtual tours, and advertising on social networks. Paradoxically, viewing digital reproductions of artwork on a computer screen can no longer be considered a “non-ecological” condition, but rather an alternative, increasingly widespread, way of experiencing art. Therefore, it is worthwhile investigating whether digital reproductions of artworks are as effective as museum originals in producing a satisfying aesthetic experience. Some prior research has already explored the influence of the *physical* context on art evaluation, comparing the aesthetic experiences in laboratory settings vs. real museums. While evaluations of objective features (symmetry, complexity, etc.) remain relatively consistent across the two contexts, the hedonic dimensions of art experiences notably differ between original museum artworks and their reproductions. In fact, original artworks in museums are liked more (Locher et al., 1999; Locher and Dolese, 2004; Brieber et al., 2014; Grüner et al., 2019; Szubielska et al., 2021a; Szubielska and Imbir, 2021) and viewed longer than digital reproductions in the lab (Brieber et al., 2014; for a review, see Pelowski et al., 2017). Artworks are also remembered better and longer when they are viewed in a museum (Brieber et al., 2015b; Specker et al., 2017). This body of research underscores the profound influence of context, that, as expected by the *aesthetic experience model* (Leder et al., 2004), improves both aesthetic emotions (in terms of valence and arousal) and aesthetic judgments (in terms of comprehension and interest). These findings are in line with theoretical discussions according to which it is hard to bring out deep human emotions and cognitive responses in artificial contexts (Specker et al., 2017; Pelowski et al., 2018).

Within this framework and drawing from on our previous museum study (Castellotti et al., 2023), we developed the current study. We aim to test whether introducing additional relevant content in labels accompanying artworks improves the aesthetic experience also in the laboratory setting. To achieve this, we recorded multiple psychophysiological (heart rate, skin conductance, eye movements, and pupil diameter) and behavioral parameters (viewing time and questionnaires) of art-naïve observers while looking at digital reproductions of contemporary paintings on a computer screen after receiving either minimal or detailed information about them. Namely, in the main experimental condition, participants were provided with “*essential*” or “*descriptive*” labels (retrieved from Castellotti et al., 2023) in two consecutive separate sessions. Essential labels provided basic information about the author, title, year, and technique of the respective paintings, while descriptive labels included the same information along with detailed descriptions of both the content of the painting and the technique used.

We chose to measure the beneficial effects of descriptive over essential labels within the same participant because the within-subjects design has been proven more effective than a between-subjects design in assessing how information impacts individual art evaluation (Russell, 2003). This choice necessarily requires providing essential labels in the first session and descriptive labels in the second session, in this fixed order for all participants. In fact, by reversing this order, participants would already acquire the descriptive information from the first session, making useless a second session with essential labels. With this design though, responses to paintings in the second sessions could potentially be influenced by the double exposure. That is, the effects recorded in the second session of the experimental condition could be attributed to familiarity effects rather than to the descriptive labels. To control for this potential effect, we introduced a secondary control condition, in which a different (smaller) set of participants were exposed twice to the paintings but without having additional information, that is they were provided with “*essential*” labels in both sessions. If similar effects are found in the experimental and control conditions, it would suggest that results are influenced by double exposure rather than the type of label; otherwise, the effect observed in the experimental condition might be attributed to the introduction of additional descriptive information.

In defining the paradigm, we tried to remain as faithful as possible to the procedure carried out in the museum (Castellotti et al., 2023): we tested a comparable sample of non-expert individuals, we used the same paintings exhibited in the museum, we recorded the same subjective and objective measures with the same setup, and we kept the same design, also including the same control condition. Thanks to the similarity between the two paradigms, our results could add further insights into the comparison of real vs. digital art experience by leveraging biometric measurements, with a particular emphasis on evaluating the efficacy of informational materials in the two contexts.

## 2 Materials and methods

### 2.1 Participants

Sixty volunteers participated in this study (all Caucasian, μ = 23.1 years, SE = 0.5); 40 participants were assigned to the main experimental condition, the other 20 to the secondary control condition. Prior to data collection, participants were asked to fill out a questionnaire about their personal data, education level, art historical background, and art expertise. All the participants were university students with school-level art history backgrounds (*n* = 5 middle-school-level, *n* = 55 high-school-level). All were naïve to the purpose of the study and given written informed consent prior to participation. All participants had normal or corrected-to-normal visual acuity, did not take any type of medication, did not present any brain damage, and were free of cognitive disorders. None of them was a painter or art student and they had visited museums or art exhibitions, on average, 3 times in the last year. They were also asked to rate their preferences for different art types (abstract, contemporary, and figurative art) on a 5-point Likert scale; on average, they reported that figurative art is significantly more appreciated than contemporary art (mean score = 3.3 ± 0.1 vs. 2.8 ± 0.1; *t*-tests across subjects, *t*_(59)_ = −2.2, *p* < 0.05). The study was conducted according to the guidelines of the Declaration of Helsinki and approved by the local ethics committee (“Commissione per l’Etica della Ricerca,” University of Florence, 2 November 2022, No. 229).

### 2.2 Setup

Pupil and gaze data were recorded by means of a wearable eye tracking headset (Pupil Core from Pupil Labs, Berlin, Germany), composed of two eye cameras (200 Hz) and a world camera (60 Hz). The device was USB-connected to an Acer computer 5 running dedicated software (Pupil Capture, version 3.5.7) that enabled real-time data capture, camera recording, and calibration routines. A wearable wireless device (like a smartwatch) equipped with high-quality data sensors (E4 wristband from Empatica Inc., Boston, MA, USA) was used to acquire electrodermal activity (EDA, 4 Hz) and heart rate (HR, computed in spans of 10 s) measures. The internal memory of E4 allowed us to record data continuously during the session (about 30 min per participant). Stimuli were displayed on AGOC gaming 27G2U/BK monitor (27-inch, 100 Hz, 1,920 × 1,080 pixels resolution), subtending 61° × 34° of visual angle at a viewing distance of 85 cm, using the Psychophysics Toolbox extensions (Brainard, 1997; Kleiner et al., 2007) for Matlab (2018b version; Mathworks, Natick, MA, USA). Participants’ responses to questionnaire items were entered on a computer keyboard by the experimenter.

### 2.3 Stimuli

The stimuli used in this experiment consisted of eight contemporary abstract paintings, the same used in our previous experiment (Castellotti et al., 2023). Original artworks were exposed in the “Roberto Casamonti Collection”,^1^ a modern and contemporary art (XX–XXI century) private museum in Florence. The museum provided us with high-quality digital reproductions that were resized, without modifying proportions, to have either a width or a height of 1,002 pixels (the other side ranged from 612 to 900 pixels); this allowed us to present stimuli that subtended similar visual angles of the paintings in the museum (on average, 21° × 15°). To see all paintings, see Supplementary Table 1.

### 2.4 Procedure

**FIGURE 1:**
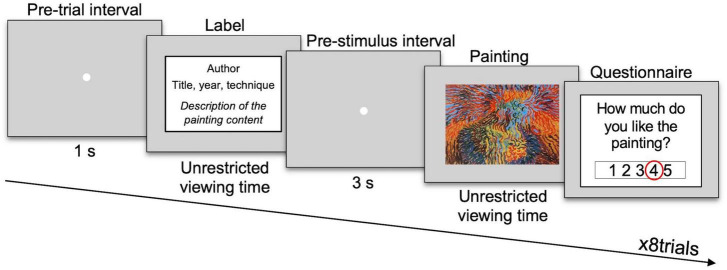
Experimental procedure. Schematic representation of a trial with a descriptive label template. The painting in the figure is copyright-free; it has been shown only for illustrative purposes and it is not part of the set of stimuli used here. Copyright laws prohibit the reproduction of the paintings used as stimuli in this experiment, but they can be viewed at the links reported in Supplementary Table 1.

We created a computer-based version of the exhibition by presenting digital reproductions of the artworks and labels in the same order as they were seen in the museum (Castellotti et al., 2023). For a schematic representation of the experimental procedure see Figure 1. Each trial started with the display of a white fixation point in the center of a gray screen for 1 s (pre-trial interval). This was followed by the presentation of one label (black-times new roman font letters presented on a 1,200 × 675 pixels white rectangular patch to mimic a paper sheet). Then, the fixation point was presented again for 3 s (pre-stimulus interval), followed by the presentation of the painting in the center of the screen. Participants could freely choose to stop reading the labels and viewing the paintings by pressing the space bar on the keyboard. Following the same paradigm used in the museum, after each stimulus, participants were required to answer several questions. The questionnaire required the participants to score on a 5-points Likert scale the following items: complexity, comprehensibility, title informativeness, positive emotions, negative emotions, liking, interest, and curiosity about seeing other works by the same artist. Additionally, participants were asked to indicate whether the paintings and the authors were familiar or unfamiliar to them.

### 2.5 Conditions

As in the previous study (Castellotti et al., 2023), both the experimental and control conditions consisted of two sessions performed on two different days, at least 4 weeks apart. At the beginning of each session, participants wore the instruments, and the eye tracker was calibrated with an 8-points natural-features calibration routine. In the experimental condition, participants were presented with *essential labels* in the first session and with *descriptive labels* in the second session. Essential labels only report minimal details about the paintings (i.e., author, title, year, and technique), whereas descriptive labels report additional information (i.e., author, title, year, technique, and description of the painting’s content and technique). In the control condition, participants were provided with the same essential labels in the first and the second sessions. To read all essential and descriptive labels, see Supplementary Table 1.

### 2.6 Data processing

*Post hoc* power analyses, to test the retrospective power of the observed effects with sample sizes and parameter estimates derived from our data sets, were performed deriving the effect size from *R*-squared (*R*^2^) (G*Power 3 software; Erdfelder et al., 2009). Given the challenges in directly calculating power for non-parametric tests, an approximation method using an equivalent parametric test – ANOVA (Cohen, 1988) – was employed. Accordingly, the analyses were performed on an assumed effect size *f*^2^ of 0.5, an alpha level of 0.05, and a sample size of 40 and 20 participants.

To check for the normality of data distributions, Shapiro–Wilks tests were performed for each parameter (see Supplementary Table 2). Since most of these tests revealed deviations from normality, non-parametric statistical tests were used as main analyses. Additionally, to facilitate meaningful comparisons with the results obtained in our previous museum study (Castellotti et al., 2023), we also conducted parametric tests. That is, to compare viewing times, EDA, HR, and pupillary responses (described in the following sections) between the essential and descriptive label sessions, and between the two control sessions with essential labels, we employed both Generalized Additive Mixed Models (GAMM) and two-way ANOVAs. These models incorporated two factors: *session* (two levels: first vs. second session) and *condition* (two levels: experimental vs. control condition). In the GAMM analysis participants and artworks were included as random effects. Given that computing effect sizes for non-parametric statistical tests, such as the GAMM used in our study, is not straightforward (Baguley, 2012), to provide an understanding of the effect of each factor in our models, we reported the corresponding slope coefficients (β). In the ANOVA, *session* was included as a within-subjects factor and *condition* as a between-subjects factor. This supplementary analysis allows us to report eta squared statistics (η^2^) as effect sizes.

#### 2.6.1 Viewing time

For each participant, we calculated the reading time of each label and the viewing time of each painting, as the difference (in seconds) between the stop viewing decided by the observer (key press) and the onset of the label/stimulus appearance. To compare viewing times between the essential and descriptive label sessions, and between the two control sessions with essential labels, we employed both a GAMM and a two-way ANOVA. *p*-Values obtained from *post hoc* analyses were adjusted using the Bonferroni corrections.

The reading time of each label from each participant was correlated to the viewing time of the corresponding following painting (Pearson linear-correlation coefficient).

#### 2.6.2 Psychophysiological measurements

For each participant, eye tracking and physiological data were continuously recorded from the start to the end of each session. Raw data from the wristband and the eye tracker were extracted in.csv format and synchronized through an ad-hoc procedure in Matlab (R2020b version; Natick, MA, USA: The MathWorks Inc.). In the data cleaning process, six inadequate recordings of the experimental condition and five of the control condition were excluded from the final analysis due to technical issues (e.g., incomplete recordings, saving failures, failed synchronization between devices, and eye-tracker calibration errors). The final sample of 49 recordings, nevertheless, yielded a rich sample of collected data, with an average recording duration of approximately 20 min per subject, totaling nearly 17 h of data recordings.

##### 2.6.2.1 Electrodermal activity and heart rate

For each observer, in each trial, the baseline EDA and HR values were calculated by averaging their values recorded over the last second of the pre-stimulus interval. This baseline value was then subtracted from the trial recording, to obtain the EDA and HR variations induced by each painting. The average value and root mean square error (RMSE) of EDA and HR during viewing time were calculated for each normalized trace induced by each painting for each participant. GAMM and a two-way ANOVA were used to compare values and RMSEs of EDA and HR responses. *p*-Values obtained from *post hoc* analyses were adjusted using the Bonferroni corrections.

##### 2.6.2.2 Pupillary response

Right and left pupil diameters were averaged, and the resulting value was transformed from pixels to millimeters. Calibration was attained by measuring the instrument’s recording of a 4 mm artificial pupil, positioned at the approximate location of the subject’s left eye. For each observer, in each trial, a baseline pupil diameter was calculated by averaging the pupil diameter recorded over the last 500 ms of the pre-stimulus interval. For measuring pupil size variations induced by paintings, this baseline value was then subtracted from each recording of that observer over the whole period of stimulus viewing. The average pupil value of each normalized trace induced by each painting was calculated for each participant. To compare pupil responses, GAMM and a two-way ANOVA were performed. *p*-Values obtained from *post hoc* analyses were adjusted using the Bonferroni corrections.

##### 2.6.2.3 Eye movements

The technology used here does not allow the automatic mapping of gaze position as it is recorded through a head-centered camera subjected to head movements. Therefore, the large amount of recorded videos (∼17 h), required an intense frame-by-frame manual coding of fixations locations (e.g., as in Pelowski et al., 2018; Rainoldi et al., 2018; Di Giovanni, 2020; Reitstätter et al., 2020; Kühnapfel et al., 2023). First, we divided each painting into three zones with different eccentricity from the center of the canvas: central zone 0°–7°, close periphery 7°–14°, and far periphery 14°–21° (see Supplementary Figure 1A). Then, we performed an additional subdivision into four zones: upper-left, upper-right, lower-left, and lower-right quadrants (see Supplementary Figure 1B), to explore potential left-right and up-down asymmetries in fixations distributions. All video recordings were extracted using the Pupil Player software and each position of the gaze shown in the videos was manually converted to a position in one of the zones. Then we counted how many times each area had been watched by each participant in the first and second sessions of the experimental and control conditions. Since the number of fixations in the two sessions is different, the proportion of fixations in each area (with respect to the total number of fixations in that session) was calculated for each session. The proportions of fixations in each zone in the descriptive vs. essential label and between the two sessions with essential labels have been compared with paired-sample Wilcoxon signed-rank tests across paintings.

#### 2.6.3 Questionnaire scores

For each painting, the scores assigned to each questionnaire item were averaged across participants. To compare average scores between the essential and the descriptive label conditions, and between the two control sessions with essential labels, paired-sample Wilcoxon signed-rank tests across paintings were performed. The effect size of the differences between conditions was estimated by Rank-Biserial correlation (rrb) with 95% confidence intervals (CI). Individual scores of each painting were also correlated with the corresponding viewing time, EDA, HR, and pupil response (Pearson linear-correlation coefficient).

## 3 Results

*Post hoc* power analyses (see section “Materials and methods”) returned a power of 0.9 for the sample sizes of both the experimental (40 participants) and control conditions (20 participants), confirming the appropriate statistical power of the following results.

### 3.1 Viewing time

Table 1 shows the medians of reading and viewing times of each label and painting in the experimental and control conditions. In the experimental condition, the median reading time of essential labels is naturally shorter than that of longer descriptive labels (6.8 ± 0.3 vs. 23.5 ± 0.8 s, SE across participants), while in the control condition, the median time dedicated to the essential labels in the two conditions is almost the same (7.8 ± 0.4 vs. 5.5 ± 0.4 s, SE across participants).

**TABLE 1:** Reading time of labels and viewing time of each painting in the experimental and control conditions.

	Experimental condition				Control condition			
	Essential label		Descriptive label		First essential label		Second essential label	
Paintings	Reading (s)	Viewing (s)	Reading (s)	Viewing (s)	Reading (s)	Viewing (s)	Reading (s)	Viewing (s)
Ebla	9.0 ± 0.5	12.0 ± 1.9	24.0 ± 1.4	14.5 ± 1.4	8.0 ± 0.5	15.5 ± 1.6	7.5 ± 0.6	12.0 ± 1.2
Empreinte d’un nu (f7)	10.0 ± 0.5	13.0 ± 1.4	27.0 ± 1.3	12.0 ± 1.3	9.5 ± 0.7	15.0 ± 1.3	8.0 ± 1.1	8.0 ± 0.9
L-1-75	8.0 ± 0.5	11.0 ± 1.1	23.0 ± 0.9	10.0 ± 1.1	8.0 ± 0.5	12.0 ± 1.0	6.5 ± 0.6	7.0 ± 1.1
Empremta de cos	8.0 ± 0.4	10.5 ± 1.6	26.5 ± 0.8	10.5 ± 1.2	9.0 ± 0.7	14.5 ± 1.5	5.0 ± 0.7	8.0 ± 0.7
Femme	5.5 ± 0.8	11.0 ± 1.4	20.0 ± 0.6	9.0 ± 1.6	6.0 ± 0.5	11.0 ± 1.2	4.0 ± 0.4	7.0 ± 1.2
Still life	5.5 ± 0.5	12.5 ± 1.5	20.0 ± 0.9	10.0 ± 1.8	6.0 ± 0.4	12.0 ± 2.2	4.0 ± 1.0	8.5 ± 2.1
Canto dal mare	5.0 ± 0.5	10.0 ± 1.2	21.0 ± 1.1	8.5 ± 1.0	6.0 ± 0.5	11.0 ± 1.5	4.5 ± 0.5	7.5 ± 1.1
Cuore pompeiano	6.0 ± 0.4	9.0 ± 1.5	25.0 ± 1.0	8.0 ± 2.0	7.0 ± 1.0	12.0 ± 1.6	5.5 ± 0.5	7.0 ± 1.1

Data in the table are medians of times with SE across participants.

**FIGURE 2:**
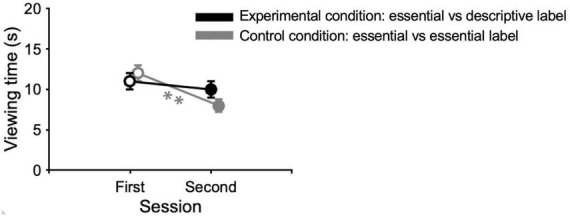
Viewing time. Data points represent medians of viewing times of paintings. Errors are 95% confidence intervals. Significance levels refer to GAMM *post hoc* comparisons: **p < 0.01.

More interesting results come from the comparison of the viewing times that observers dedicated to the paintings after reading different labels. Analyses on viewing times show a significant effect of the session [GAMM: *t* = −5.2, *p* < 0.001, β = −4.8; ANOVA: *F*(1,58) = 17.5, *p* < 0.001, η^2^ = 0.05] and of the interaction between sessions and conditions [GAMM: *t* = 2.8, *p* < 0.01, β = 3.2; ANOVA: *F*(1,58) = 4.42, *p* < 0.05, η^2^ = 0.01], but there is no significant difference between conditions [GAMM: *t* = −1.5, *p* > 0.05, β = −2.7, ANOVA: *F*(1,58) = 1.29, *p* > 0.05, η^2^ = 0.02]. Indeed, in the experimental condition, the time spent viewing the paintings is the same in the first and in the second session, so descriptive labels do not seem to influence viewing time (*post hoc* comparisons; GAMM: *t* = 2.4, *p* > 0.05; ANOVA: *t* = 1.8, *p* > 0.05; Figure 2). Instead, in the control condition, viewing time is significantly longer in the first than in the second session, both with essential labels (*post hoc* comparisons; GAMM: *t* = 5.2, *p* < 0.01; ANOVA: *t* = 3.8, *p* < 0.01; Figure 2). These results suggest that, although the descriptions did not lead to an increase in viewing time, their presence might have prevented a decrease in viewing time due to a repetition of the same experience in the second session. Analyses also confirm that viewing times in the first sessions of the experimental and control conditions are comparable, as expected because in the first session of both conditions participants were subjected for the first time to the same experience: painting observation after reading an essential label (*post hoc* comparisons; GAMM: *t* = −0.6, *p* > 0.05; ANOVA: *t* = −0.1, *p* > 0.05).

While viewing times do not differ much across different paintings (as can be appreciated in Table 1), our data show large differences across participants (see Supplementary Figure 2). For example, when considering the first sessions with essential labels, the median of times spent by each participant viewing different paintings ranged from 4 to 38 s (Supplementary Figure 2). In an attempt to explain these individual differences, we measured the correlations between participants viewing time and their art experience and preferences, reported before data collection, but no correlations were found.

Finally, we correlated the reading time of each label with the viewing time of the corresponding painting in all sessions (see Supplementary Figure 3). There is a positive significant correlation in the first sessions with essential labels (aggregating experimental and control conditions; *r* = 0.3, *p* < 0.001; Supplementary Figure 3A). The correlation is also significant in the second session of the experimental condition with descriptive labels (*r* = 0.3, *p* < 0.001; Supplementary Figure 3B), but it is not significant in the second session of the control condition with essential labels (see Supplementary Figure 3C).

### 3.2 Electrodermal activity and heart rate

**FIGURE 3:**
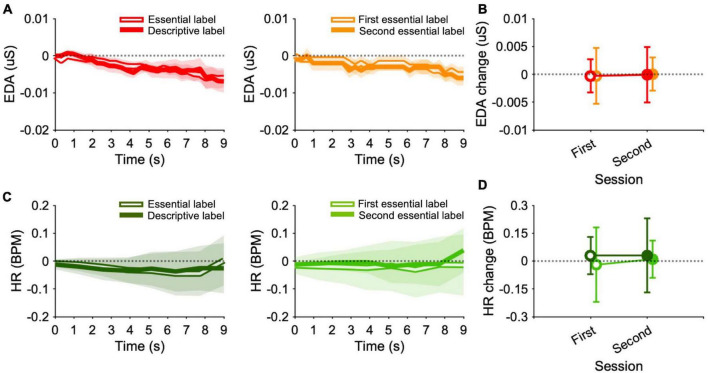
Electrodermal activity and heart rate. **(A)** Line graphs show EDA response over time averaged across paintings and participants for the experimental (left panel) and control condition (right panel). Plots are obtained first by averaging point-by-point individual normalized traces induced by each painting (only time points including at least 15 experimental participants, or 5 control participants were averaged). Then, average traces for each painting were averaged together (only time points that were recorded for all paintings in both labels were averaged). **(B)** Data points represent medians of EDA responses. Errors are 95% confidence intervals. **(C)** Line graphs show HR response over time averaged across paintings and participants for the experimental (left panel) and control condition (right panel). Plots are obtained as described in panel **(A)**. **(D)** Data points represent medians of HR responses. Errors are 95% confidence intervals.

Electrodermal activity responses over time averaged across paintings and participants of the experimental and control conditions are reported in Figure 3A. In all sessions and conditions, EDA values are comparable with the baseline levels. Analyses on EDA values show that there are no significant differences between conditions [GAMM: *t* = −0.03, *p* > 0.05, β = −0.0002; ANOVA: *F*(1,47) = 0.17, *p* > 0.05, η^2^ = 0.003], sessions [GAMM: *t* = 0.07, *p* > 0.05, β = 0.0002; ANOVA: *F*(1,47) = 0.003, *p* > 0.05, η^2^ = 0.0], and their interaction [GAMM: *t* = −0.16, *p* > 0.05, β = 0.0006; ANOVA: *F*(1,47) = 0.03, *p* > 0.05, η^2^ = 0.0; see Figure 3B]. Analyses performed on EDA variability (RMSE), also reveal no significant differences between conditions [GAMM: *t* = −0.16, *p* > 0.05, β = −0.001; ANOVA: *F*(1,47) = 0.0004, *p* > 0.05, η^2^ = 0.0], sessions [GAMM: *t* = 0.50, *p* > 0.05, β = 0.002; ANOVA: *F*(1,47) = 0.09, *p* > 0.05, η^2^ = 0.0], and their interaction [GAMM: *t* = −0.34, *p* > 0.05, β = −0.001; ANOVA: *F*(1,47) = 0.03, *p* > 0.05, η^2^ = 0.0].

Heart rate responses over time averaged across paintings and participants of the experimental and control conditions are reported in Figure 3C. HR values are compatible with baseline values for all sessions of all conditions. Analyses provide no significant differences for HR values between conditions [GAMM: *t* = 0.6, *p* > 0.05, β = 0.2: ANOVA: *F*(1,47) = 0.6, *p* > 0.05, η^2^ = 0.006], sessions [GAMM: *t* = 0.3, *p* > 0.05, β = 0.05; ANOVA: *F*(1,47) = 0.04, *p* > 0.05, η^2^ = 0.0], and the interaction between them [GAMM: *t* = −0.8, *p* > 0.05, β = −0.1; ANOVA: *F*(1,47) = 0.6, *p* > 0.05, η^2^ = 0.008; see Figure 3D]. Similar results were found for HR RMSE concerning the condition factor [GAMM: *t* = −0.2, *p* > 0.05, β = −0.02; ANOVA: *F*(1,47) = 0.6, *p* > 0.05, η^2^ = 0.02], session factor [GAMM: *t* = −1.4, *p* > 0.05, β = −0.08; ANOVA: *F*(1,47) = 0.05, *p* > 0.05, η^2^ = 0.007] and the interaction [GAMM: *t* = 1.1, *p* > 0.05, β = 0.06; ANOVA: *F*(1,47) = 0.6, *p* > 0.05, η^2^ = 0.004].

Overall, these findings show no evidence of EDA and HR variations during painting viewing. There is also no evidence that their level and variability are affected by the presence of informative descriptions or subjected to familiarity effects.

### 3.3 Pupillary response

**FIGURE 4:**
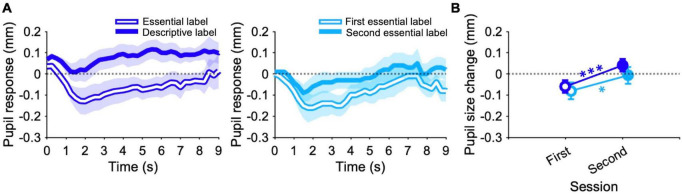
Pupillary response. **(A)** Line graphs show pupil response over time averaged across paintings and participants for the experimental (left panel) and control condition (right panel). Plots are obtained first by averaging point-by-point individual normalized traces induced by each painting (only time points including at least 15 experimental participants, or 5 control participants were averaged). Then, average traces for each painting were averaged together (only time points that were recorded for all paintings in both labels were averaged). **(B)** Data points represent medians of pupil responses. Errors are 95% confidence intervals. Significance levels refer to GAMM *post hoc* comparisons: *p < 0.05, ***p < 0.001.

Pupil responses over time averaged across paintings and participants of the experimental and control conditions are reported in Figure 4A. In the experimental condition, the pupil is more dilated with descriptive than with essential labels during the whole paintings viewing time (Figure 4A, left panel). A similar trend, although with a smaller difference, is observed in the control condition, where the pupil is always slightly more dilated in the second than in the first session with essential labels (Figure 4A, right panel). These differences are confirmed by the analyses performed on pupil responses (Figure 4B). Statistical analyses reveal a significant effect of the session [GAMM: *t* = 2.6, *p* < 0.01, β = 0.07; ANOVA: *F*(1,47) = 47.8, *p* < 0.001, η^2^ = 0.1]. Indeed, in the experimental condition, pupil size is larger after descriptive than after essential labels (*post hoc* comparisons; GAMM: *t* = −6.9, *p* < 0.001; ANOVA: *t* = −8.3, *p* < 0.001). In the control condition with essential labels, pupil is also larger in the second than in the first session (*post hoc* comparisons; GAMM: *t* = −2.6, *p* < 0.05; ANOVA: *t* = −2.8, *p* < 0.05). Besides, the pupil size in the second session of the experimental condition is significantly larger than that in the second session of the control condition (*post hoc* comparisons; GAMM: *t* = −4.5, *p* < 0.001; ANOVA: *t* = −2.7, *p* < 0.05). Overall, these results indicate that both the description and the second exposure induce a larger pupil size than that obtained in the first session where participants see paintings with essential labels, but the effect caused by providing additional information seems to be larger than that induced by the familiarity for the paintings.

### 3.4 Eye movements

**FIGURE 5:**
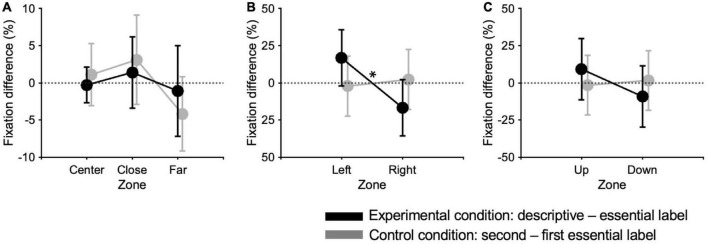
Eye movements. Differences of spatial distribution of fixations across three canvas eccentricities **(A)**, between left and right sides **(B)**, and between upper and lower canvas zones **(C)**. Data points represent the difference of the percentage of fixations between the first and second sessions in the experimental and control conditions. Errors are SE across paintings. Significance levels refer to paired-sample Wilcoxon signed-rank test: *p < 0.05.

In the analysis of eye movements, we firstly focused on the distribution of fixations across the different eccentricities of the canvas (Supplementary Figure 1A). In the experimental condition, when observers are provided with essential labels vs. descriptive labels, they dedicated 9.8% (±1.2 SE) vs. 9.5% (±1.1 SE) of fixations on the center, 37.2% (±2.9 SE) vs. 38.6% (±1.9 SE) on the close periphery, and the 53% (±3.7 SE) vs. 51.9% (±2.4 SE) on the far periphery. In the control condition, when observers are provided with the first essential labels vs. the second essential labels, they dedicated 5.8% (±1.8 SE) vs. 7.4% (±2.7 SE) of fixations on the center, 37.4% (±4.2 SE) vs. 41.4% (±2.8 SE) on the close periphery, and the 35.4% (±3.3 SE) vs. 34.6% (±4.5 SE) on the far periphery. The differences in the percentage of fixations across these canvas zones between different label types in the experimental and control conditions are reported in Figure 5A. None of these differences are statistically significant (all paired-sample Wilcoxon signed-rank tests between fixations in different eccentricities yield *p* > 0.5).

Additionally, we explored the distribution of fixations between left/right sides and between up/down zones of the canvas (Supplementary Figure 1B). In the experimental condition, when observers are provided with essential vs. descriptive labels, they directed 52.3% (±3.4 SE) vs. 68.9% (±3.4 SE) of fixations to the left side, and 47.7% (±3.5 SE) vs. 31.1% (±3.4 SE) to the right side. In the control condition, when observers are provided with the first essential labels vs. the second essential labels, they directed 53.2% (±3.6 SE) vs. 51.0% (±3.9 SE) of fixations to the left side, and 46.8% (±3.6 SE) vs. 49.0% (±3.9 SE) to the right side. The differences in the percentage of fixation between left and right zones for different label types in the experimental and control conditions are reported in Figure 5B. Statistical analyses show that the number of fixations between the left and right side resulted significantly different only in the descriptive label condition (paired-sample Wilcoxon signed-rank tests: *W* = 3, *p* < 0.05).

Regarding up/down fixations distributions, in the experimental condition for essential vs. descriptive labels, observers directed 68.7% (±2.9 SE) vs. 77.8% (±4.2 SE) of fixations to the upper zone, and 31.3% (±2.9 SE) vs. 22.1% (±4.2 SE) to the lower zone. In the control condition, for first vs. second essential labels, observers directed 80.7% (±3.3 SE) vs. 79.1% (±3.9 SE) of fixations to the upper zone, and 19.3% (±3.3 SE) vs. 20.2% (±3.9 SE) to the lower zone. The differences in the percentage of fixation between up and down zones for different label types in the experimental and control conditions are reported in Figure 5C. No significant differences emerged in the number of fixations between the upper and lower zones.

### 3.5 Questionnaire scores

**FIGURE 6:**
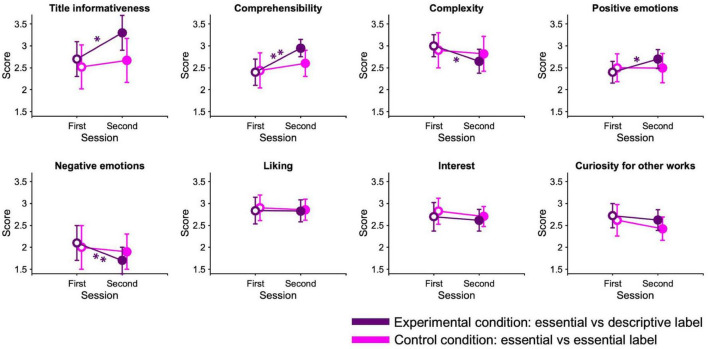
Questionnaire scores. Data points represent average scores that participants attributed to the different items of the questionnaire, averaged across paintings. Errors are 95% confidence intervals. Significance levels refer to paired-sample Wilcoxon signed-rank test: *p < 0.05; **p < 0.01.

In the experimental condition, for some questionnaire items, participants’ subjective judgments of paintings changed after reading a descriptive label, whereas, in the control condition, no significant differences emerge between the sessions for any items (Figure 6). In fact paired-sample Wilcoxon signed-rank tests across paintings shows a significant effect of descriptions on *title informativeness* (*W*(7) = 1, *p* < 0.05, rrb = −0.94, 95% CI [−0.99, −0.76]), *comprehensibility* (*W*(7) = 0, *p* < 0.01, rrb = −1.00, 95% CI [−1.00, −1.00]), and *complexity* (*W*(7) = 35, *p* < 0.05, rrb = 94.00, 95% CI [0.76, 0.99]), indicating that descriptive labels helped the global understanding of artworks. In addition, descriptive labels significantly increase *positive emotions* (*W*(7) = 1, *p* < 0.05, rrb = −0.97, 95% CI [−0.99, −0.26]) and decrease *negative emotions* (*W*(7) = 36, *p* < 0.01, rrb = 1.00, 95% CI [1.00, 1.00]). There is no evidence of a change between the two sessions of the experimental condition for the remaining items.

None of the questionnaire responses to the paintings were correlated with viewing time or physiological responses to the corresponding artworks (all *p* > 0.05).

## 4 Discussion

Numerous studies suggests that educational materials accompanying artworks, such as informative labels, play crucial role in enhancing the aesthetic experience (Falk and Dierking, 2000; Reitstätter et al., 2020). However, some museums have reduced or even eliminated explanations and labels (Trione, 2017, 2023; Conti, 2022) in the attempt to make the experience “more emotional and less cultural-driven” (Pekarik, 2004; Sitzia, 2018). Scientifically assessing the impact of informative materials on the perception and understanding of artworks can guide museum institutions in improving the quality of visitors’ experience, whether they are at the museum in person or accessing art online.

To this end, in our previous study, we focused on the controversial context of a modern art museum, investigating the comparative effects of essential vs. descriptive labels on the cognitive and emotional experience of visitors with limited prior knowledge of art. Overall, our findings showed that naïve people benefit significantly from receiving additional detailed information about artworks (Castellotti et al., 2023).

In the current work, motivated by the growing prevalence of digital media in the art world, we investigated whether the benefits of introducing additional relevant descriptions can be obtained by presenting the same labels and paintings on a computer screen. By recording the same objective and subjective parameters and adopting the same experimental paradigm as in our previous work (Castellotti et al., 2023), we can also provide a reliable comparison between the aesthetic experience in the laboratory and the museum setting.

First, we observe no significant differences in the amount of time participants spent viewing artworks when they were provided with either an essential or a descriptive label. This outcome aligns with a prior laboratory study, which found that the time allocated to viewing paintings remained consistent regardless of the type of information provided, whether it was factual or contextual (Lin and Yao, 2018). Most of the other lab studies about titles and explanations used certain given times for stimuli presentation (Leder et al., 2006; Bubić et al., 2017; Szubielska et al., 2018; Ganczarek et al., 2022) or they did not record this variable (Millis, 2001; Mullennix and Robinet, 2018), therefore we cannot make comparisons. Crucially, our results on viewing time diverge from those found in the museum setting (Castellotti et al., 2023), where we observed that descriptions increased the time spent on visualizing artworks, but they are still consistent with the findings of other museum studies (Smith et al., 2017). On the other hand, participants of the control condition spent significantly less time observing the paintings during the second session when essential labels were provided, which mirrors our findings in the museum setting, although with a smaller effect size (Castellotti et al., 2023). This suggests that double exposure to the artworks without additional information may lead to a lower engagement in the aesthetic experience in both contexts. In summary, in the laboratory setting, descriptions do not seem to encourage participants to extend their art-viewing time, but they appear to prevent the decline in interest that can result from repeated exposure to the same artworks.

Regardless of the type of label used, in the laboratory setting, participants spent significantly less time viewing the artworks compared to the museum environment (Castellotti et al., 2023), with durations less than half of those recorder previously. This striking disparity aligns with findings from previous research (Brieber et al., 2014).

The time devoted to reading digital captions in the lab is notably lower (almost half) than that allocated to reading paper-based labels in the museum of identical length and content (Castellotti et al., 2023), whereas identical reading times have been reported in both contexts (as in Brieber et al., 2014). This finding prompts us to speculate that our young adult observers may be more accustomed to reading texts on a computer screen than on traditional paper.

Consisted with findings from other studies (Heidenreich and Turano, 2011; Brieber et al., 2014; Smith et al., 2017), we observe substantial individual variability in viewing time among participants, suggesting that personal characteristics may influence viewing behavior. However, here viewing time does not significantly correlate with personal art preferences and experience (collected before the experiment), probably because variability in art expertise amongst participants is negligible. Conversely, variability in viewing times of paintings is relatively smaller, suggesting that time spent may not depend on specific features of individual artworks.

Finally, we found a positive correlation between reading time of labels and viewing times of paintings when observers first view the paintings or when they view them for the second time with detailed description. This finding implies that the amount of attention allocated to the labels somehow influences the attention to the following artworks, provided that the didactic materials are novel for the observers.

Another significant effect of introducing descriptive labels emerges in participants’ responses to the questionnaire. Providing additional information about artworks enhances their comprehension and subsequently reduces the perceived complexity. It also elevates the prominence of the artworks’ titles and fosters positive emotions. On the other hand, there is no evidence that descriptive labels influence the observers’ appreciation, as their liking and interest levels seem not change with the type of labels used. It seems therefore that non-expert individuals have an improved understanding of abstract art and experience more positive emotions after exposure to informative materials, but this does not necessarily lead to an increase in their overall hedonic evaluation of the paintings. Prior research has yielded contradictory findings in this regard, also depending on the experimental design adopted (Russell, 2003). Our results are not in line with the studies showing that information makes artworks more pleasant (Millis, 2001; Russell, 2003; Swami, 2013; Gerger and Leder, 2015; Szubielska et al., 2021a), but still confirm other research (Russell and Milne, 1997; Lin and Yao, 2018; Szubielska and Imbir, 2021). It is important to note that all these studies utilize didactic materials with varying degrees of informativeness. Given that our texts primarily focus on abstract, formal, and technical aspects, rather than the direct meaning of the artworks, it is possible that our novice participants struggled to relate meaningfully with the concepts presented in the artworks. It is even plausible that specific art training, which facilitates the development of aesthetic fluency (Smith and Smith, 2006), may be necessary to appreciate such complex artworks. There is no evidence that double exposure to paintings without providing additional information modifies questionnaire responses. This may indicate that subjective judgments are not affected by familiarity with the paintings and that the effects observed in the experimental condition are due to the descriptions, and not to repeated ratings. The same results emerged in our museum work (Castellotti et al., 2023) and other lab studies (Russell, 2003).

Based on earlier works, we would expect a higher degree of appreciation and interest in the real-world setting than in the controlled laboratory environment (Locher et al., 2001; Brieber et al., 2014, 2015b; Szubielska et al., 2021a). Surprisingly, we found that the ratings given to digital reproductions are similar to those assigned to real artworks displayed in the museum (Castellotti et al., 2023). It is worth noting that in both our studies participants consistently provide low scores, never surpassing 3 out of 5. This might suggest that abstract artists do not prioritize beauty in their work, but instead aim to provoke thoughts and foster ambiguity (Szubielska and Imbir, 2021). Consequently, these artworks may not necessarily be aesthetically pleasing, even in a museum context. We can also speculate that the widespread (unavoidable) reliance on computers for everyday activity in the pandemic area, during which the study took place, has contributed to a normalization of the digital encounter with art. As a result, our young (digital-natives) participants may evaluate virtual experiences on a par with in-person ones. The absence of differences between the real and the laboratory contexts also emerged in other recent works (Brieber et al., 2015a; Reitstätter et al., 2020).

Another noteworthy result is the absence of a correlation between questionnaire ratings and the corresponding behavioral and psychophysiological responses to a given painting. For example, drawing from previous research findings, one might have anticipated that higher levels of appreciation and understanding would be associated with increased viewing times for artworks and labels (Leder et al., 2006; Swami, 2013; Brieber et al., 2014), but this was not the case. This is especially surprising when considering the use of descriptive labels, which are supposed to assist viewers in understanding the technique, theme, and intention of the artist. On the other hand, other researchers failed to find this correlation, claiming that, since aesthetic judgments depend on numerous factors, time spent looking at artworks may not be a reliable predictor (Heidenreich and Turano, 2011; Bullot and Reber, 2013). Aesthetic appreciation ratings do not correlate with pupillary responses either (cf. Kuchinke et al., 2009). The lack of correlations between subjective judgments and biometric measurements, consistent with Castellotti et al. (2023) results, is probably due to the fact all our participants provided low ratings for liking and interest.

Turning to the psychophysiological findings, first, we observe that pupil size is larger when participants viewed artworks with descriptive labels compared to essential labels. Moreover, a similar, albeit smaller, effect also surfaces when observers viewed the same paintings a second time without additional information. It is worth noting that in our previous work pupil responses changed exclusively upon detailed descriptions (Castellotti et al., 2023). From these results, we can infer that the pupil responds not only to the acquisition of new information but also, to some extent, to familiarity with the stimuli (Kuchinke et al., 2009; Massaro et al., 2012; Castellotti et al., 2020, 2021). Note that the effect size of the pupillary results obtained here are even larger than that obtained in the museum, maybe due to a more controlled experimental environment. Pupillary dilation typically indicates increased arousal due to emotional involvement. However, based on this assumption, we might anticipate concurrent changes in skin conductance and heart rate, as these are strongly influenced by the sympathetic nervous system (Sequeira et al., 2009; Gernot et al., 2018). Nevertheless, neither galvanic skin response nor heart rate seem to be affected by different types of labels or double exposure. Note that EDA results do not align with our previous work, in which we observed an increase in average skin conductance with both descriptions and familiarity (Castellotti et al., 2023). Consequently, we can speculate that the pupil response in our study is influenced by high-level factors beyond mere arousal levels, such as cognitive and memory effort or art appreciation (even though we have already discussed that the items related to these factors do not correlate with pupil responses).

Regardless of the type of label, we can note that EDA and HR remain relatively stable in response to the presentation of the paintings on the computer screen, In contrast, observers exhibited a steep increase in skin conductance when began to view the artworks in the museum (Castellotti et al., 2023). This seems then to confirm that experiencing live art evokes a stronger reaction (Szubielska et al., 2021a). Since pupil diameter is strongly influenced by ambient light, luminance of the stimuli, and their background, we cannot make meaningful comparisons of absolute values of pupil sizes between the real museum context and the laboratory setting.

Regarding eye movements, in our previous work, we found a more spread distribution of visual attention with descriptive labels, which encouraged participants to move their eyes toward the periphery of the canvas (Castellotti et al., 2023). Here, there is no evidence that the accompanying text influence the gaze pattern across different eccentricities (Lin and Yao, 2018). This analysis is rather coarse, comparing fixations only within the central, near-peripheral, and far-peripheral regions of the paintings. However, given that our eye movement analysis relies on manual coding of fixations, a finer analysis dividing the paintings into smaller zones could have introduced consistent subjective biases based on the experimenter’s criterion.

In the present work, we also explored the possible presence of a left-right asymmetry in fixation distribution, which might be expected given the lateralization of spatial attention (Sosa et al., 2010). Indeed, we found a preference for the left side of the canvas when providing descriptive labels, suggesting an increase in attention to the following painting, and therefore to a left-biased asymmetry in its exploration.

It important to note that, since we observed the descriptive label effects in a condition where paintings were also seen for the second time, it is possible that participants were excited/physiologically aroused by learning new additional information about artworks that they had seen before. In other words, the label-based effects we found could be conditional on paintings that participants are already familiar with. To address this possibility, further studies implementing different experimental designs should be conducted in laboratory and museum settings.

Overall, this and previous studies using descriptive labels, demonstrate the relevance of providing meaningful information regarding artworks, for viewers’ understanding of such art and for provoking enhanced psychophysiological responses also in the “artificial” context. This issue is very important in modern experimental aesthetics and may have important practical implications and applications, for example, when deciding how to present artworks in museums as well as on digital platforms.

Like many studies in empirical aesthetics, our sample is composed of recruited (psychology) students, whose real intrinsic motivation to see the artworks is uncertain. While this approach ensures a relatively homogeneous sample for age, socioeconomic status, and education, it also allows for the control of the level of art expertise. Nevertheless, further studies should be conducted with participants spontaneously engage in digital art consumption to enhance the ecological validity and broaden our understanding of the virtual art experience and its comparison with the in-person experience. Our experimental paradigm, not allowing participants to engage in typical behaviors commonly seen in both in-person and digital art experiences, such as revisiting preferred paintings or switching between reading labels and viewing artworks, constitutes another limitation of the current study. Additionally, it is important to remark that the two types of labels used here not only vary in the amount of information about the painting but also in their length, visual appearance, and the cognitive effort they demand from the reader. This implies that the effects observed with informative labels might not solely stem from the artwork description itself, rather it is possible that similar effects could arise using other kinds of lengthy texts. Investigating how different types of text (relevant, irrelevant, emotional, neutral, etc.) impact psychophysiological responses during picture viewing would be intriguing, although it is beyond the scope of our current research. Furthermore, one might question why we did not include a no-label condition as a baseline; this was not feasible in our previous museum experiment because the paintings were already accompanied by essential labels (author, title, year, and technique) as part of the exhibition’s standard setup. Consequently, we had to use the essential labels as the baseline condition initially, and we opted to maintain consistency with this approach in the current study.

## 5 Conclusion

Relying on the evidence that, in the nowadays digital world, the experience of art outside a museum is no longer a “non-ecological” situation, here we assessed the effectiveness of introducing additional informative materials on the enjoyment of digital reproduction of contemporary art paintings. While laboratory studies in empirical aesthetics have traditionally focused on behavioral data such as viewing times and preference ratings, we complemented these measurements with objective biometric parameters, such as pupil responses, eye movements, heart rate, and electrodermal activity. The integration of both behavioral and psychophysiological data lead to a complex picture of different findings hardly to interpret in a unique way, particularly when compared with our previous work in the museum (Castellotti et al., 2023). In both the laboratory and museum contexts, after receiving detailed descriptions, observers find the content less complex and more arousing, and their pupil size increases. However, unlike in the real context, in the lab, they spend the same amount of time inspecting artworks with essential or descriptive labels, following a similar gaze pattern, and they do not exhibit changes in electrodermal activity or heart rate. The largest difference between the two contexts is the smaller amount of viewing time dedicated to digital reproductions vs. original paintings viewed in person. Subjective judgments (e.g., appreciation, interest, and emotions), however, seem to be comparable in the two contexts.

Overall, while the contextual environment affects the aesthetic experience, our findings suggest that some of the beneficial effects of informative material can also be observed in digital media outside of the museum context.

As a final consideration, it is worthwhile noting that visual art attendance has not been replaced by digital alternatives, unlike other forms of art and expression, such as music. Indeed, in-person attendance to art museums and galleries is still the preferential modality whether virtual internet resources are used only to complement the real visit (Marty, 2008). Although this research, as well as many others, brings some evidence in favor of an enhanced aesthetic experience in the real context, what drives such individual and public investments in in-person experience despite the current availability of digital alternatives must be still investigated.

## Data availability statement

The datasets presented in this study can be found in online repositories. The names of the repository/repositories and accession number(s) can be found below: all data are available from the Zenodo database (https://doi.org/10.5281/zenodo.11485735).

## Ethics statement

The studies involving humans were approved by the Commissione per l’Etica della Ricerca, University of Florence, 2 November 2022, No. 229. The studies were conducted in accordance with the local legislation and institutional requirements. The participants provided their written informed consent to participate in this study.

## Author contributions

SC: Conceptualization, Data curation, Formal analysis, Investigation, Methodology, Resources, Software, Validation, Visualization, Writing – original draft, Writing – review & editing. OD’A: Data curation, Formal analysis, Investigation, Methodology, Software, Visualization, Writing – original draft, Writing – review & editing. MD: Conceptualization, Funding acquisition, Project administration, Resources, Supervision, Writing – original draft, Writing – review & editing.

## Footnotes

1
https://collezionerobertocasamonti.com
