# Supplementary material for: Effectiveness of labels in digital art experience: psychophysiological and behavioral evidence
Source: Front Psychol. 2024 Jul 1;15:1342667. doi: 10.3389/fpsyg.2024.1342667 (PMC11248719; doi:10.3389/fpsyg.2024.1342667)
Supplement: Supplementary file 1 [file Table_1.DOCX]

**Supplementary Table 1. Paintings and their essential and descriptive labels.** First column: web link to the paintings used as stimuli (in the same order as presented to participants). Second column: essential labels read by the participants in the first experimental session and by control participants in the two control sessions. Third column: descriptive labels read by the participants in the second experimental session. In each row, labels in Italian (the language used in the experiment) are reported in regular style; labels translated in English are reported in italics style.

| **Painting link** | **Essential label** | **Descriptive label** |
| --- | --- | --- |
| <https://collezionerobertocasamonti.com/?portfolio=giulio-paolini#&gid=1&pid=1> | Giulio Paolini,  EBLA, 1976-1977,  Smalto e collage su tavola.  *Giulio Paolini,*  *EBLA, 1976-1977,*  *Collage on acrylic painted board.* | Giulio Paolini, EBLA, 1976-1977, Smalto e collage su tavola. L’opera è stata realizzata nel 1976 dal Giulio Paolini, esponente del movimento “concettuale”, una corrente nata intorno al 1960 che si concentra sul problema dell’ideazione di un manufatto artistico. “Ebla” prende nome dall’antica città sorta nella Siria settentrionale, celebre per il suo patrimonio archeologico: l’artista rappresenta un via colonnata, posta al centro di una superficie che imita l’effetto del marmo. In tal modo, lo spettatore è invitato ad immergersi nel senso della storia e della memoria.  *Giulio Paolini, EBLA, 1976-1977, Collage on acrylic painted board. The work was created in 1976 by Giulio Paolini, an exponent of the "conceptual" movement, born around 1960 which focuses on the problem of conceiving an artistic artefact. "Ebla" takes its name from the ancient city built in northern Syria, famous for its archaeological heritage. The artist represents a colonnaded street, placed in the center of a surface that imitates the effect of marble. In doing so, the viewer is invited to immerse himself in the sense of history and memory.* |
| <https://collezionerobertocasamonti.com/?portfolio=yves-klein-2#&gid=1&pid=1> | Yves Klein,  MARQUE DE FEU –  EMPREINTE D’UN NU (F7), 1961,  Cartone bruciato su pannello.  *Yves Klein,*  *MARQUE DE FEU – EMPREINTE D'UN NU (F7), 1961,*  *Burnt cardboard mounted on board.* | Yves Klein, MARQUE DE FEU – EMPREINTE D’UN NU (F7), 1961, Cartone bruciato su pannello. L’artista francese Yves Klein (famoso per le sue creazioni monocrome di colore blu) sperimentò una grande varietà di linguaggi, di materiali e di tecniche. L’opera che vediamo appartiene a una serie in cui egli cerca di esaltare gli effetti spontanei degli elementi naturali (come l’acqua e il fuoco). Nel 1961, egli si cimentò nell’uso di una torcia industriale che orientava su una particolare tela resistente alle fiamme, con l’aiuto di assistenti dotati di idranti. Si tratta di azioni che l’artista compie senza poterne pienamente controllare il risultato.  *Yves Klein, MARQUE DE FEU – EMPREINTE D'UN NU (F7), 1961, Burnt cardboard mounted on board. The French artist Yves Klein (famous for his monochrome blue creations) experimented with a great variety of languages, materials and techniques. The work we see belongs to a series in which he tries to enhance the spontaneous effects of natural elements (such as water and fire). In 1961, he tried his hand at using an industrial torch which he directed on a particular flame-resistant canvas, with the help of assistants equipped with water cannons. These are actions that the artist performs without being able to fully control the result.* |
| <https://collezionerobertocasamonti.com/?portfolio=conrad-marca-relli#&gid=1&pid=1> | Conrad Marca-Relli,  L-1-75, 1975,  Collage e tecnica mista su tela.  *Conrad Marca-Relli,*  *L-1-75, 1975,*  *collage and mixed media on canvas.* | Conrad Marca-Relli, L-1-75, 1975, Collage e tecnica mista su tela. L’artista italo-americano Marca-Relli appartiene alla schiera dei grandi protagonisti dell’arte del Novecento: fu amico di Jackson Pollock e di Alberto Burri e lavorò costantemente tra New York e Roma. Dopo una stagione di arte figurativa, si accostò ai linguaggi dell’Espressionismo astratto, sperimentando gli effetti poetici del colore e dei materiali. In questa opera egli mostra il suo interesse per la tecnica del collage, combinando elementi che dialogano tra loro nella forma e nella diversità della superficie.  *Conrad Marca-Relli, L-1-75, 1975, collage and mixed media on canvas. The Italian-American artist Marca-Relli belongs to the group of the great protagonists of 20th century art. He was friend with Jackson Pollock and Alberto Burri and worked constantly between New York and Rome. After a season of figurative art, he approached the languages ​​of “Abstract Expressionism”, experimenting with the poetic effects of color and materials. In this work he shows his interest in the collage technique, combining elements with different shapes and surfaces.* |
| <https://collezionerobertocasamonti.com/?portfolio=antoni-tapies#&gid=1&pid=2> | Antoni Tàpies,  EMPREMTA DE COS, 1982,  Tecnica mista su carta intelata.  *Antoni Tàpies,*  *EMPREMTA DE COS, 1982,*  *media on a paper laid down on canvas.* | Antoni Tàpies, EMPREMTA DE COS, 1982, Tecnica mista su carta intelata. Tra i movimenti artistici sviluppatisi dopo la Seconda guerra mondiale, molto importante fu il cosiddetto “Informale”: una corrente che pone la sua massima attenzione al procedimento esecutivo dell’opera, dunque al gesto compiuto dall’artista. Antoni Tàpies fu tra i massimi interpreti di questo linguaggio. In questa opera possiamo riconoscere la forza del “segno” lasciato sulla tela: una traccia che testimonia l’energia interiore dell’artista, la sua spontaneità. Ciò che è importante per lo spettatore non è ritrovare in queste macchie una “forma riconoscibile”, ma una dichiarazione di libertà espressiva.  *Antoni Tàpies, EMPREMTA DE COS, 1982, media on a paper laid down on canvas. The so-called "Informal" artistic movement was very important among those developed after the Second World War. The movement placed its utmost attention on the executive procedure of the work, therefore on the gesture made by the artist. Antoni Tàpies was one of the greatest interpreters of this language. In this work we can recognize the strength of the "sign" left on the canvas: a trace that testifies the artist's inner energy and spontaneity. What is important for the viewer is not to find a "recognizable form" in these color stains, but a declaration of freedom of expression.* |
| <https://collezionerobertocasamonti.com/?portfolio=joan-miro#&gid=1&pid=1> | Joan Miró,  FEMME, 1977-1978,  Olio su pannello.  *Joan Miró,*  *FEMME, 1977-1978,*  *Oil on panel.* | Joan Miró, FEMME, 1977-1978, Olio su pannello. Il dipinto risale alla fase più tarda dell’opera del catalano Joan Mirò, tra i principali esponenti del Surrealismo. L’interesse dell’artista per il sogno si manifesta in questo caso attraverso forme reali che vengono alterate e deformate, proprio come avviene in un’esperienza inconscia. Le pennellate dense e corpose delineano con tratti molto semplici la forma di un corpo femminile, che viene vivacizzato dall’uso di macchie di colore (verde, bianco, rosso, giallo).  *Joan Miró, FEMME, 1977-1978, Oil on panel. The painting dates back to the later phase of the work of the Catalan artist, one of the main exponents of “Surrealism”. The artist's interest in dreams is manifested in this case through real forms that are altered and deformed, just as it happens in an unconscious experience. The dense and full-bodied brushstrokes outline the shape of a female body with very simple strokes, which is enlivened by the use of splashes of color (green, white, red, yellow).* |
| <https://collezionerobertocasamonti.com/?portfolio=francesco-clemente#&gid=1&pid=1> | Francesco Clemente,  STILL LIFE, 1986,  Acrilico su tela.  *Francesco Clemente,*  *STILL LIFE, 1986,*  *Acrylic on canvas.* | Francesco Clemente, STILL LIFE, 1986, Acrilico su tela. Francesco Clemente, che vive a New York ed ha maturato una importante esperienza in India, è un esponente della Transavanguardia italiana, un movimento che promuove un ritorno al linguaggio figurativo. L’immagine rappresenta una natura morta, cioè un genere molto comune nella pittura del passato. Ad essa, Clemente attribuisce dei colori acidi, con forti contrasti, ed utilizza un punto di vista dall’alto che schiaccia la figura, con un effetto che la allontana dalla realtà.  *Francesco Clemente, STILL LIFE, 1986, Acrylic on canvas. Francesco Clemente, who lives in New York and has gained important experience in India, is an exponent of the “Italian Transavantgarde”, a movement that promotes a return to the figurative language. The picture represents a still life, that is, a very common genre in the painting of the past. For this subject, Clemente uses acid colors with strong contrasts, and uses an overhead view, that crushes the figure, with the effect of alienation from reality.* |
| <https://collezionerobertocasamonti.com/?portfolio=nicola-de-maria#&gid=1&pid=1> | Nicola De Maria,  CANTO DAL MARE, 1989,  Olio su tela.  *Nicola De Maria,*  *CANTO DAL MARE, 1989,*  *oil on panel.* | Nicola De Maria, CANTO DAL MARE, 1989, Olio su tela. Nicola De Maria è uno dei principali esponenti del movimento della Transavanguardia. La sua pittura, che può essere di piccole o grandi dimensioni, ha come obiettivo principale quello di ispirare poeticamente il pubblico. Il suo linguaggio è intenzionalmente semplice e somiglia per certi aspetti all’innocenza della creatività infantile. In Canto del mare, i piccoli tratti ondulati, alternati a segni orizzontali e verticali, trasmettono un senso di armonia e di leggerezza. I segni ricurvi ricordano l’aspetto di un mare leggermente mosso.  *Nicola De Maria, CANTO DAL MARE, 1989, oil on panel. Nicola De Maria is one of the major exponents of the “Transavantgarde” movement. His paintings, which can be small or large, have the main objective of poetically inspiring the public. His language is intentionally simple and somehow resembles the innocence of childish creativity. In “Canto del mare”, the small wavy strokes, alternating with horizontal and vertical signs, convey a sense of harmony and lightness. The curved signs recall the appearance of a slightly rough sea.* |
| <https://collezionerobertocasamonti.com/?portfolio=jim-dine#&gid=1&pid=1> | Jim Dine,  CUORE POMPEIANO, 1985,  Carbone, olio e acrilico su carta.  *Jim Dine,*  *POMPEIAN HEART, 1985,*  *Carbon, oil and acrylic on paper.* | Jim Dine, CUORE POMPEIANO, 1985, Carbone, olio e acrilico su carta. Jim Dine è uno dei più importanti artisti americani del Novecento, vicino alla Pop Art e affermatosi con il movimento New Dada, che si caratterizza per la combinazione di tecniche tradizionali con oggetti della vita quotidiana. In Cuore pompeiano, l’artista dipinge l’immagine di un cuore, da tutti associata al sentimento dell’amore e all’affettività. Questo oggetto simbolo, usato nei fumetti e in pubblicità, viene dipinto su un fondo azzurro con alcune creature acquatiche che ricordano un paesaggio marino. Questa insolita associazione rende l’immagine misteriosa, stimolando la fantasia dello spettatore.  *Jim Dine, POMPEIAN HEART, 1985, Carbon, oil and acrylic on paper. Jim Dine is one of the most important American artists of the twentieth century, close to “Pop Art”. He established himself with the “New Dada” movement, which is characterized by the combination of traditional techniques with everyday objects. In “Pompeian Heart”, the artist paints the image of a heart, associated to the feeling of love and affection. This symbolic object, used in comics and in advertising, is painted on a blue background with some aquatic creatures resembling a seascape. This unusual association makes the picture mysterious, and it stimulates the imagination of the viewer.* |

**Supplementary Table 2. Results of Shapiro Wilk test for normality.** P-values below 0.05 indicate that the data significantly deviate from a normal distribution.

|  | **Experimental condition** | | **Control condition** | |
| --- | --- | --- | --- | --- |
|  | **Essential label** | **Descriptive label** | **First essential label** | **Second essential label** |
| **Viewing times** | W=0.8, *p* < 0.001 | W=0.7, *p* < 0.001 | W=0.9, *p* < 0.001 | W=0.8 *p* < 0.001 |
| **EDA values** | W=0.5, *p* < 0.001 | W=0.6, *p* < 0.001 | W=0.6, *p* < 0.001 | W=0.7, *p* < 0.001 |
| **EDA RMSE** | W=0.3, *p* < 0.001 | W=0.4, *p* < 0.001 | W=0.5, *p* < 0.001 | W=0.4, *p* < 0.001 |
| **HR values** | W=0.9, *p* < 0.001 | W=0.7, *p* < 0.001 | W=0.8, *p* < 0.001 | W=0.9, *p* < 0.001 |
| **HR RMSE** | W=0.6, *p* < 0.001 | W=0.6, *p* < 0.001 | W=0.6, *p* < 0.001 | W=0.7, *p* < 0.001 |
| **Pupil values** | W=0.9, *p* < 0.001 | W=0.9, *p* < 0.001 | W=0.9, *p* < 0.001 | W=0.9, *p* < 0.001 |

**
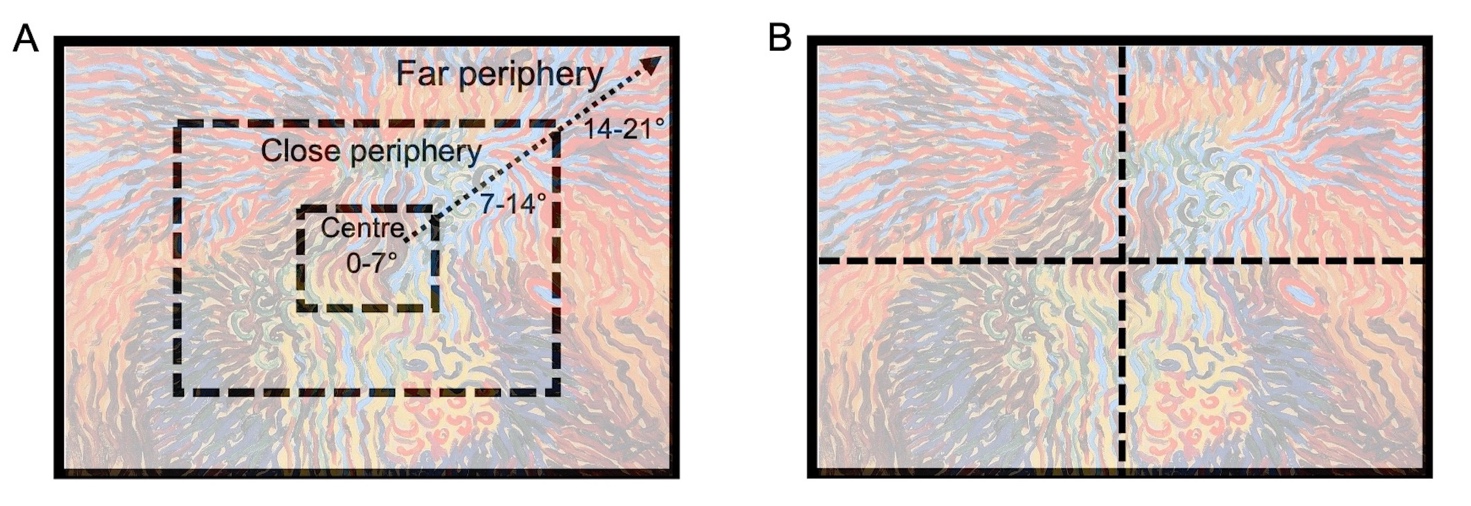
**

**Supplementary Figure 1. Schematic representations of subdivision of canvas zones for eye movements analyses. (A)** Subdivision of each painting into three zones with different eccentricities from the center of the canvas. **(B)** Subdivision of each painting into four zones: up-left, up-right, down-left, down-right.

**
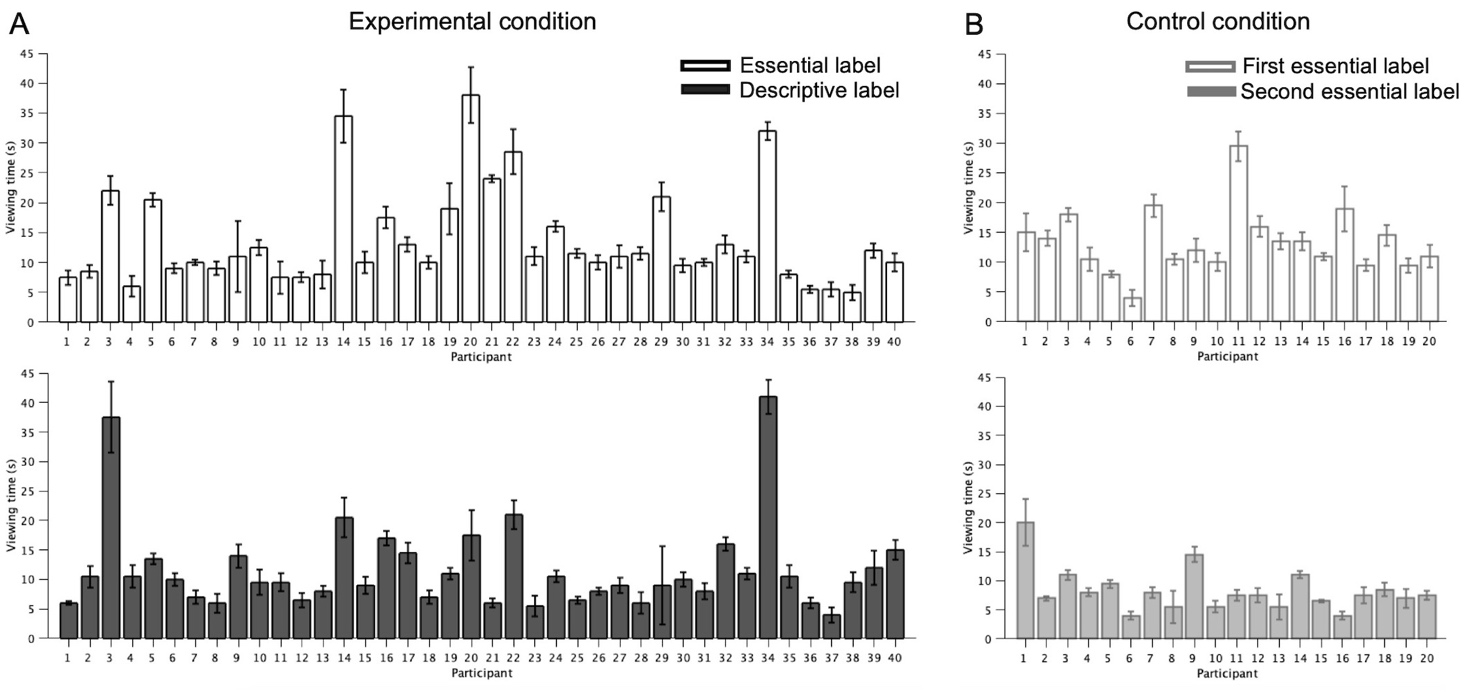
**

**Supplementary Figure 2. Individual viewing times.** Bars represent median of times spent viewing the different paintings by each participant in the experimental **(A)** and control condition **(B)**. Errors are SE across paintings.


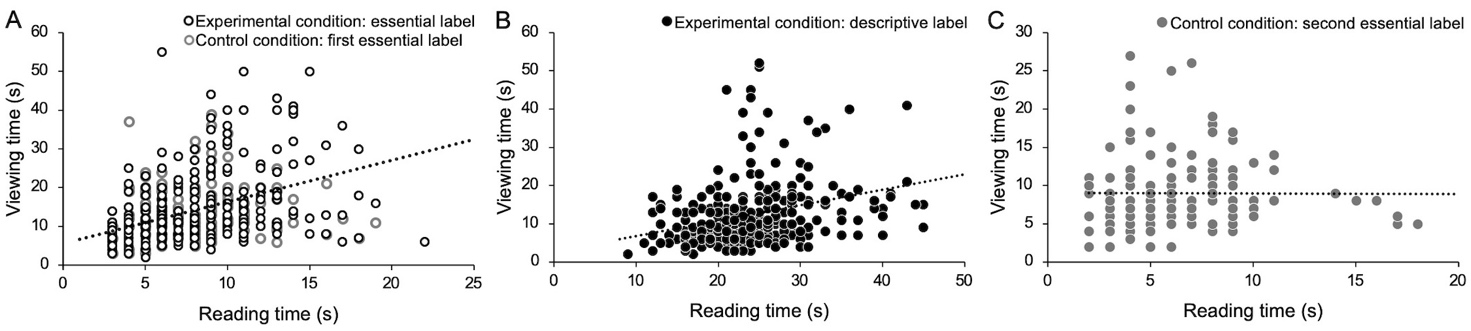


**Supplementary Figure 3. Correlation between reading and viewing times. (A)** First sessions with essential labels of experimental and control conditions. **(B)** Second sessions with descriptive labels of the experimental condition. **(C)** Second sessions with essential labels of the control condition.
